# CircRHOT1 mediated cell proliferation, apoptosis and invasion of pancreatic cancer cells by sponging miR‐125a‐3p

**DOI:** 10.1111/jcmm.15572

**Published:** 2020-07-22

**Authors:** Sunkai Ling, Yanru He, Xiaoxue Li, Mingyue Hu, Yu Ma, Yuan Li, Zipeng Lu, Shanshan Shen, Bo Kong, Xiaoping Zou, Kuirong Jiang, Peilin Huang

**Affiliations:** ^1^ Medical School of Southeast University Nanjing Jiangsu China; ^2^ Pancreas Center The First Affiliated Hospital with Nanjing Medical University Nanjing Jiangsu China; ^3^ Department of Gastroenterology, the Affiliated Drum Tower Hospital Nanjing University Medical School Nanjing Jiangsu China; ^4^ Department of Surgery Klinikum rechts der Isar School of Medicine Technical University of Munich (TUM) Munich Germany

**Keywords:** ceRNA, circRHOT1, E2F3, miR‐125a‐3p, pancreatic cancer

## Abstract

Pancreatic cancer patients are asymptomatic at early stages and leading to late diagnoses. Additionally, pancreatic cancer easily metastasizes and is resistant to radiotherapy and chemotherapy. Therefore, it is critical to understand the underlying molecular mechanisms involved in pancreatic cancer to develop more efficient diagnostic and treatment strategies. In this study, we demonstrated that circRHOT1 was overexpressed in pancreatic cancer tissues and cell lines, and it was found to directly bind to miR‐125a‐3p, acting as an endogenous sponge to inhibit its activity. Knockdown of circRHOT1 expression significantly inhibited proliferation as well as invasion, and it promoted apoptosis of pancreatic cancer cells via the regulation of E2F3 through the targeting of miR‐125a‐3p. Taken together, our results showed that circRHOT1 plays critical roles in regulating the biological functions of pancreatic cancer cells, suggesting that circRHOT1 may serve as a potential diagnostic marker and therapeutic target for patients with pancreatic cancer.

## 1 BACKGROUND

It is well known that most patients who are at an early stage of pancreatic cancer are asymptomatic, and currently, there is no specific screening method for pancreatic cancer; therefore, most patients are not diagnosed until the disease has reached an advanced or a late stage. Pancreatic cancer easily metastasizes and is resistant to radiotherapy and chemotherapy.^1^, ^2^, ^3^ Therefore, it is critical to understand the underlying molecular mechanisms involved in pancreatic cancer to develop more efficient diagnostic and treatment strategies.

Circular RNAs are a class of special endogenous non‐coding RNAs that have a closed continuous loop structure, lack 5'‐3' polarity and contain no poly‐A tail.^4^ Studies have shown that some circRNAs are abundant and stable in mammalian cells.^5^, ^6^, ^7^ There is increasing evidence indicating that circRNAs play a role in the development of multiform types of disease,^8^, ^9^ especially in cancer, where circRNAs are often aberrantly expressed.^10^, ^11^, ^12^ Due to their structural features, circRNAs can act as microRNA sponges, RNA‐binding protein sequestering agents and nuclear transcriptional regulators, meaning they can regulate gene expression at both the transcriptional and post‐transcriptional levels.^13^ Several types of circRNAs have been shown to play key roles in pancreatic cancer. One study demonstrated that hsa_circ_0005397 is up‐regulated in pancreatic ductal adenocarcinoma (PDAC).^14^ hsa_circ_0005397 is derived from the RHOT1gene and has been termed circRHOT1.The circRHOT1 expression was related to the pancreatic cancer cell proliferation, invasion and migration.^15^ However, the function and potential mechanism of circRHOT1 in pancreatic cancer remain largely unknown.

In this study, we found that circRHOT1 was up‐regulated in pancreatic cancer tissues and cell lines and that it directly binds to miR‐125a‐3p, acting as an endogenous sponge to inhibit miR‐125a‐3p activity. circRHOT1 mediates a regulatory pathway critical for the regulation of proliferation, invasion, the cell cycle and apoptosis of pancreatic cancer cells, which suggests that circRHOT1 might be a reasonable diagnostic and therapeutic target.

## 2 MATERIALS AND METHODS

### 2.1 Population

Twenty‐eight pairs of PDAC fresh‐frozen tissues, and adjacent non‐tumour tissues between December 2016 and July 2017 were generously given from Pancreas Biobank, The First Affiliated Hospital with Nanjing Medical University, which is a part of Jiangsu Biobank of Clinical Resource. None of the patients received radiotherapy, chemotherapy or targeted therapy before surgery. RNA samples from the tissues were extracted in Department of Gastroenterology, the Affiliated Drum Tower Hospital of Nanjing University Medical School, Nanjing, Jiangsu, China. The experiments utilizing human samples were approved by the Ethical Committee of Medical Research, the Affiliated Drum Tower Hospital of Nanjing University Medical School (2016‐191‐01).

### 2.2 Cell culture

The human pancreatic cancer cell lines (PANC‐1, SW1990, COLO357 and CF‐PAC1) and the human pancreatic ductal cell line (HPDE) were purchased from GeneChem (Shanghai, China). Cells were cultured in DMEM (Gibco, Thermo Fisher Scientific, Waltham, MA, USA) which was supplemented with 10% foetal bovine serum (Gibco, Thermo Fisher Scientific) and 1% penicillin and streptomycin (Solarbio, Beijing, China), and they were maintained in a 37℃ incubator containing 5% CO_2_. The medium was replaced every 24‐48 hours according to the cell density. Cells were observed under an inverted microscope and were digested with 0.25% trypsin (Gibco, Thermo Fisher Scientific) to enable passaging of the cells when they reached 80% confluence.

### 2.3 Quantitative real‐time PCR

Total RNA was isolated using TRIzol reagent. Then, the concentration of RNA was measured using a spectrophotometer (Titertek‐Berthold Colibri). Complementary DNA was synthesized using a PrimeScript RT reagent kit (Takara Bio Inc, Dalian, China), and quantitative real‐time PCR was performed using SYBR Premix Ex Taq (Takara Bio Inc, Dalian, China). The threshold cycle (Ct) values for circRHOT1 and E2F3 were normalized against the Ct value of glyceraldehyde‐3‐phosphate dehydrogenase (GAPDH), which was an internal control, while miR‐125a‐3p was normalized against U6 snRNA, which was an internal control. The relative RNA expression values were calculated using the 2^−ΔΔCt^ method.

### 2.4 Western blot analysis

Protein was extracted using a Total Protein Extraction kit (KeyGEN Biotech, Nanjing, China), and the protein concentration was quantified using a BCA Protein Assay kit (KeyGEN Biotech). Each sample containing an equivalent amount of protein (20 µg) was separated by 10% SDS‐PAGE and transferred to polyvinylidene difluoride (PVDF) membranes. After blocking with 5% skim milk powder for 1 hour at room temperature, the PVDF membranes were incubated overnight at 4°C with a rabbit antibody against E2F3 (1:1000; Affinity Biosciences, New Jersey, USA) and a rabbit monoclonal antibody against GAPDH (1:1000) (Beyotime Biotechnology, Beijing, China). Then, the membranes were washed three times with TBS‐T buffer, which was followed by incubation with a goat anti‐rabbit secondary antibody (1:1000; Beyotime Biotechnology) for 1 hour at room temperature. Immunoreactive proteins were detected using an ECL Reagent (Affinity) and an automatic chemiluminescence image analysis system (Tanon 5200, Shanghai, China).

### 2.5 Colony formation assay

Cell culture dishes (35 mm) were used, and each dish was covered with 2 mL of complete medium and 800 cells. After culturing for 14 days at 37°C, the colonies were fixed with 4% formaldehyde for half an hour, stained with 0.1% crystal violet solution for half an hour, imaged and counted.

### 2.6 CCK‐8 assay

Cell Counting Kit‐8 (CCK‐8) assays were performed with an Enhanced Cell Counting Kit‐8 (Beyotime Biotechnology). PANC‐1 cells were seeded into 96‐well plates, and after adherence overnight, we transfected the cells according to the experimental design. Then, 10 µL of CCK‐8 solution was added to each well. After 4 hours of incubation at 37°C with 5% CO_2_, the absorbance was measured at 450 nm by a microplate reader. We collected data once a day at the same time for 4 days.

### 2.7 5‐Ethynyl‐20‐deoxyuridine assay

A Cell‐Light 5‐ethynyl‐20‐deoxyuridine (EdU) DNA Cell Proliferation kit from Donghuan (Shanghai, China) was used. PANC‐1 cells were seeded into 24‐well plates and then were transfected. When the cell density was close to 80%, the cells were incubated with serum‐free DMEM supplemented with 10 μmol/L EdU for an additional 2 hours at 37°C, and then they were fixed with 4% formaldehyde. After EdU and DNA staining for 30 minutes each, images were immediately captured. All images were obtained with an Olympus FSX100 microscope (Olympus, Tokyo, Japan). The ratio of EdU‐stained cells to Hoechst‐stained cells was used to evaluate cell proliferation.

### 2.8 RNA interference and stable transfection

Two small interfering RNAs (siRNAs) targeting the back‐splice junction of circRHOT1 (si‐circRHOT1‐1 and si‐circRHOT1‐2) were designed and synthesized by Genechem. Through the detection, si‐circRHOT1‐1 knockdown efficiency was better than that of si‐circRHOT1‐2; the sh‐circRHOT1 corresponding si‐circRHOT1‐1 was packaged into a GV248 lentiviral vector by Genechem. A miR‐125a‐3p mimic, an inhibitor and siRNAs targeting E2F3 (si‐E2F3) were designed and constructed by GenPharma. The cells transfected with the sh‐circRHOT1 lentivirus were cultured with 3 µL/mL puromycin for 4 days to generate a stably transfected cell line. Lipofectamine 3000 (Invitrogen, Carlsbad, USA) was used for siRNA and plasmid transfection.

### 2.9 Dual‐luciferase assay

Luciferase vectors with the 3‘untranslated regions（3ʹUTR) of circRHOT1 or E2F3 and their mutant versions, containing the Renilla luciferase gene (hRluc) and firefly luciferase gene (hLuc), were obtained from Genechem. 293T cells were plated in 24‐well plates and were cultured overnight. Then, luciferase vectors were cotransfected into cells with the miR‐125a‐3p mimic or a mimic NC and were incubated for 48 hours. Luciferase assays were then performed using a Dual‐Luciferase Reporter Assay System kit (E2920; Promega, Madison, USA). Firefly luciferase activity was normalized to Renilla luciferase activity and was expressed as a percentage of the control.

### 2.10 Transwell assay

Transwell chambers with Matrigel (BD Biosciences, New Jersey, USA) were used to detect cell invasion. The bottom chambers were added 500 µL of complete medium.PANC‐1 cells were digested and suspended in serum‐free medium, and 200 µL was loaded into the upper chambers (containing 6 × 10^4^ cells). After incubation at 37°C for 24 hours, the cells on the bottom of the upper chambers were fixed with 4% formaldehyde for half an hour and then stained with 0.1% crystal violet solution for half an hour; images were then collected from five different fields of each sample. The number of invasive cells was counted by ImageJ.

### 2.11 Flow cytometry

Cell apoptosis was assayed by using an Annexin V‐APC/7‐AAD apoptosis kit (MULTI, Hangzhou, China). PANC‐1 cells were collected and suspended in 1× binding buffer, and then, V‐PAC and 7‐AAD were added. After incubating in the dark for 15 minutes, the percentage of apoptotic cells was detected by flow cytometry (BD FACSCalibur, New Jersey, USA).

Cell cycle analysis was performed using a Cell Cycle Staining kit (MULTI). PANC‐1 cells were collected and suspended in DNA staining solution with 1% permeabilization solution; they were stained in the dark for 30 minutes, and then, they were detected by flow cytometry (BD FACSCalibur).

### 2.12 Statistical analysis

Comparison of data between groups is presented as the mean ± SD. Student's *t* tests, Fisher's exact tests and Mann‐Whitney tests were performed by using SPSS (v.13.0.0; SPSS Inc, Chicago, IL, USA) to determine statistical significance. *P < 0.05 was considered statistically significant, and **P < 0.01 was considered highly statistically significant.

The primer sequences are listed as follows:

**Table:** 

circRHOT1	Forward primer TGCCGTTAACAACAAGCATTC
	Reverse primer TGGAACTCTCTCTGGGGTGA
miR‐125a‐3p	Forward primer CAGGTGAGGTTCTTGGGAGC
E2F3	Forward primer TGCCTGACTCAATAGAGAGCCTAC
	Reverse primer TCCCATTGTGGTCTTGGTTGT
GAPDH	Forward primer TGTTGCCATCAATGACCCCTT
	Reverse primer CTCCACGACGTACTCAGCG
u6	Forward primer CTCGCTTCGGCAGCACA
	Reverse primer AACGCTTCACGAATTTGCGT

The relative siRNA sequences are listed as follows:

**Table:** 

si‐circRHOT1‐1	Sense CAGCAGGUUCCUCCCCGGGTT
	Antisense CCCGGGGAGGAACCUGCUGTT
si‐circRHOT1‐2	Sense ACAGCAGGUUCCUCCCCGGTT
	Antisense CCGGGGAGGAACCUGCUGUTT
si‐E2F3	Sense GCAUCCACCUCAUUAAGAATT
	Antisense UUCUUAAUGAGGUGGAUGCTT
miR‐125a‐3p mimic	Sense ACAGGUGAGGUUCUUGGGAGCC
	Antisense CUCCCAAGAACCUCACCUGUUU
miR‐125a‐3p inhibitor	Sense GGCUCCCAAGAACCUCACCUGU
si‐NC/mimic NC	Sense UUCUUCGAACGUGUCACGUTT
	Antisense ACGUGACACGUUCGGAGAATT
Inhibitor NC	Sense CAGUACUUUUGUGUAGUACAA

## 3 RESULTS

### 3.1 CircRHOT1 is increased in PDAC tissues

**FIGURE 1:**
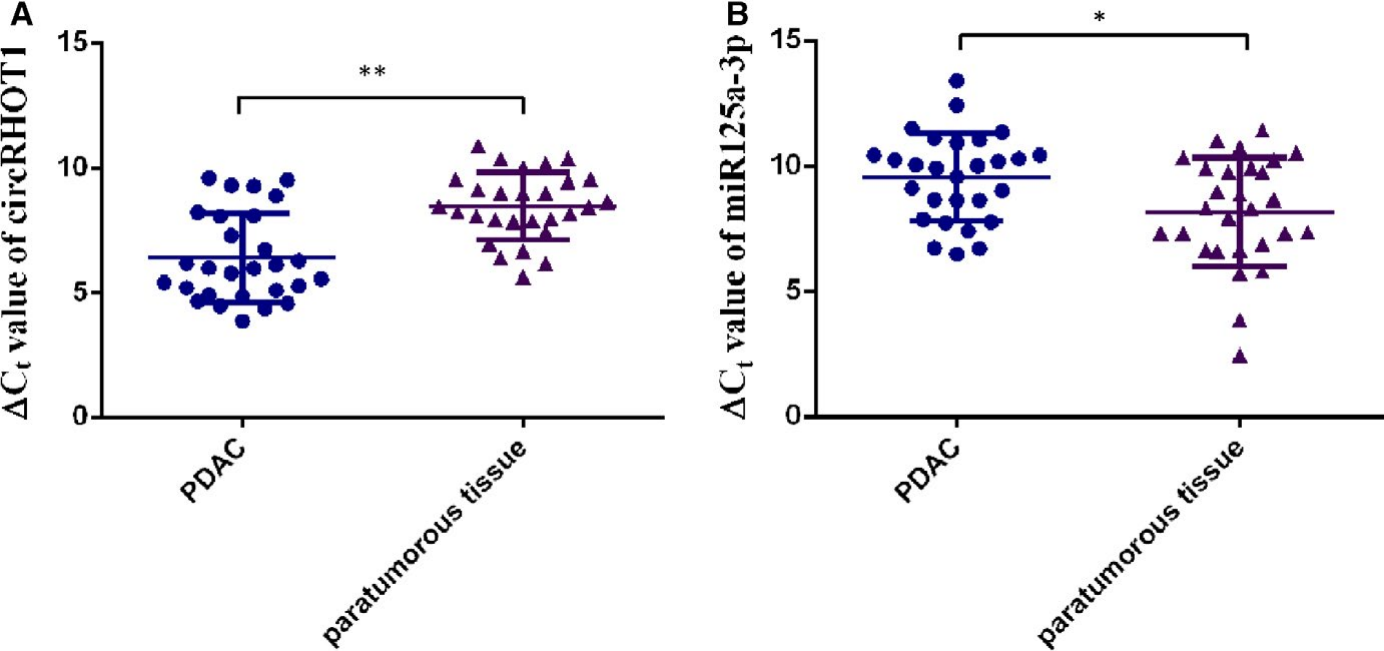
CircRHOT1 is increased in PDAC tissues. A, ΔCt value of circRHOT1 in PDAC tissues and adjacent normal tissues, as measured by RT‐qPCR. B, ΔCt value of miR‐125a‐3p in PDAC tissues and adjacent normal tissues was measured by RT‐qPCR. * P < .05, ** P < .01

Reverse transcription and quantitative real‐time PCR (RT‐qPCR) analysis was performed on 28 pairs of human PDAC specimens and their adjacent non‐cancerous tissue samples to detect the expression of circRHOT1. CircRHOT1 expression was significantly up‐regulated in PDAC compared to the related adjacent non‐cancerous tissues (Figure 1A). We then divided these cases into negative and positive groups according to the ratio of circRHOT1 expression levels. Comparison of different clinical manifestations indicated that the expression level of circRHOT1 was correlated with lymphatic metastasis, but there was no statistical significance with tumour stage and size (Table 1). We then detected the expression of miR‐125a‐3p, which has potential binding sites for circRHOT1 in 28 PDAC and adjacent tissues (Figure 1B). The expression of miR‐125a‐3p in PDAC tissues was significantly lower than that of adjacent tissues.

**TABLE 1:** Association between circRHOT1 expression and clinicopathologic features

Clinicopathologic feature	CircRHOT1 expression			*P* value
	Negative group N = 6 (%)	Positive group N = 22 (%)		
Age (y)				
65.92 ± 11.33	69.33 ± 16.48	65 ± 9.81	*T* = −0.825	0.417
Sex				
Male	4 (66.67)	15 (68.18)		0.99
Female	2 (33.33)	7 (31.82)		
Stage				
IIA	4 (66.67)	7 (31.82)		0.174
IIB or III	2 (33.33)	15 (68.18)		
Size (cm^3^)				
44.18 ± 133.78	26.2 ± 25.27	49.09 ± 150.8	*Z* = −0.617	0.654
Lymphatic metastasis				
Yes	1 (16.67)	16 (72.73)		0.022*
No	5 (83.33)	6 (27.27)		

Note

Independent‐samples *t* test was conducted to evaluate the circRHOT1 expression with age; Fisher's exact test was used to evaluate the circRHOT1 expression with sex, tumour stage and lymphatic metastasis; Mann‐Whitney test was applied to evaluate the circRHOT1 expression with tumour size.

* *P* < 0.05

### 3.2 CircRHOT1 overexpression affects the biological function of pancreatic cancer cells

**FIGURE 2:**
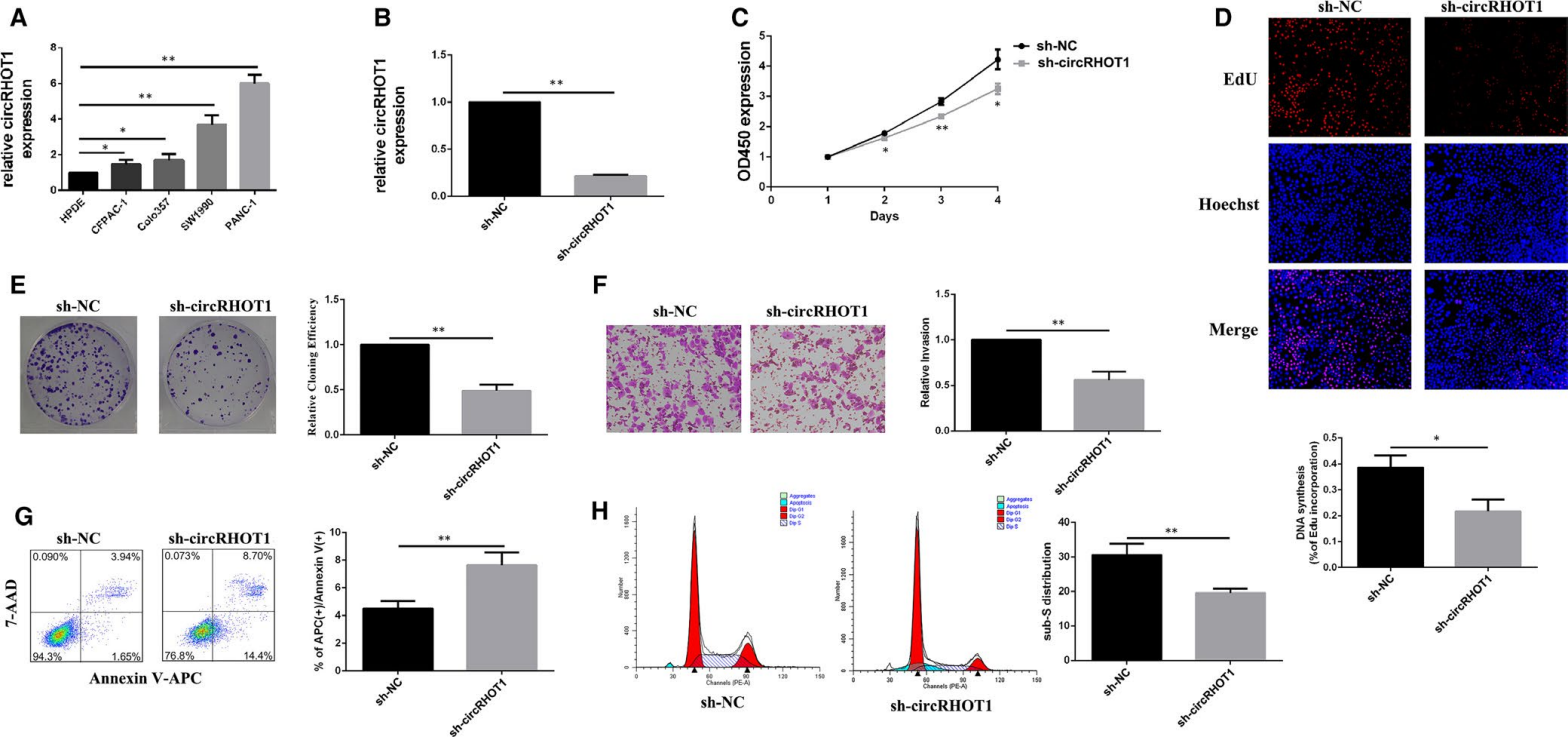
CircRHOT1 is overexpressed and affects the biological function of pancreatic cancer cells. A, Relative expression of circRHOT1 in PDAC cells and human pancreatic ductal cell line cells was measured by RT‐qPCR. B, Relative expression levels of circRHOT1 after transfection of PANC‐1 cells were measured by RT‐qPCR. C, The viability of PANC‐1 cells after transfection was detected by Cell Counting Kit‐8. D, 5‐Ethynyl‐20‐deoxyuridine assays were used to detect cell proliferation after transfection. E, Colony formation assays were used to detect clonogenic ability of PANC‐1 cells after transfection. F, Transwell assays were used to detect cell invasion capacities in PANC‐1 cells after transfection. G, Flow cytometric assays were used to detect apoptosis of PANC‐1 cells after transfection. H, Flow cytometric assays were used to observe the cell cycle after transfection.* P < .05, ** P < .01

To investigate the expression of circRHOT1 in pancreatic cancer cells, RT‐qPCR analysis was performed. We confirmed the expression level of circRHOT1 was significantly up‐regulated in PDAC cell lines compared with that of HPDE (Figure ). As the expression of circRHOT1 was the highest in PANC‐1 cells among these five cell lines, we chose PANC‐1 as our experimental cell line. To explore the function of circRHOT1 in PDAC, sh‐circRHOT1 was used to knock down the expression of circRHOT1 in PANC‐1 cells. After transfection for 72 hours, the RT‐qPCR results showed that the expression of circRHOT1 was significantly decreased in the sh‐circRHOT1 group (Figure 2B). Decreased circRHOT1 levels resulted in inhibited cell proliferation (Figure 2C and 2D) and colony‐forming capacity relative to that of the control cells (Figure 2E). Additionally, knockdown of circRHOT1 significantly suppressed the invasiveness of PANC‐1 cells (Figure 2F). Moreover, this inhibition promoted apoptosis (Figure 2G) and reduced the number of PANC‐1 cells arrested in S phase (Figure 2H).

### 3.3 MiR‐125a‐3p has a crucial role in regulating the biological function in PANC‐1 cells

**FIGURE 3:**
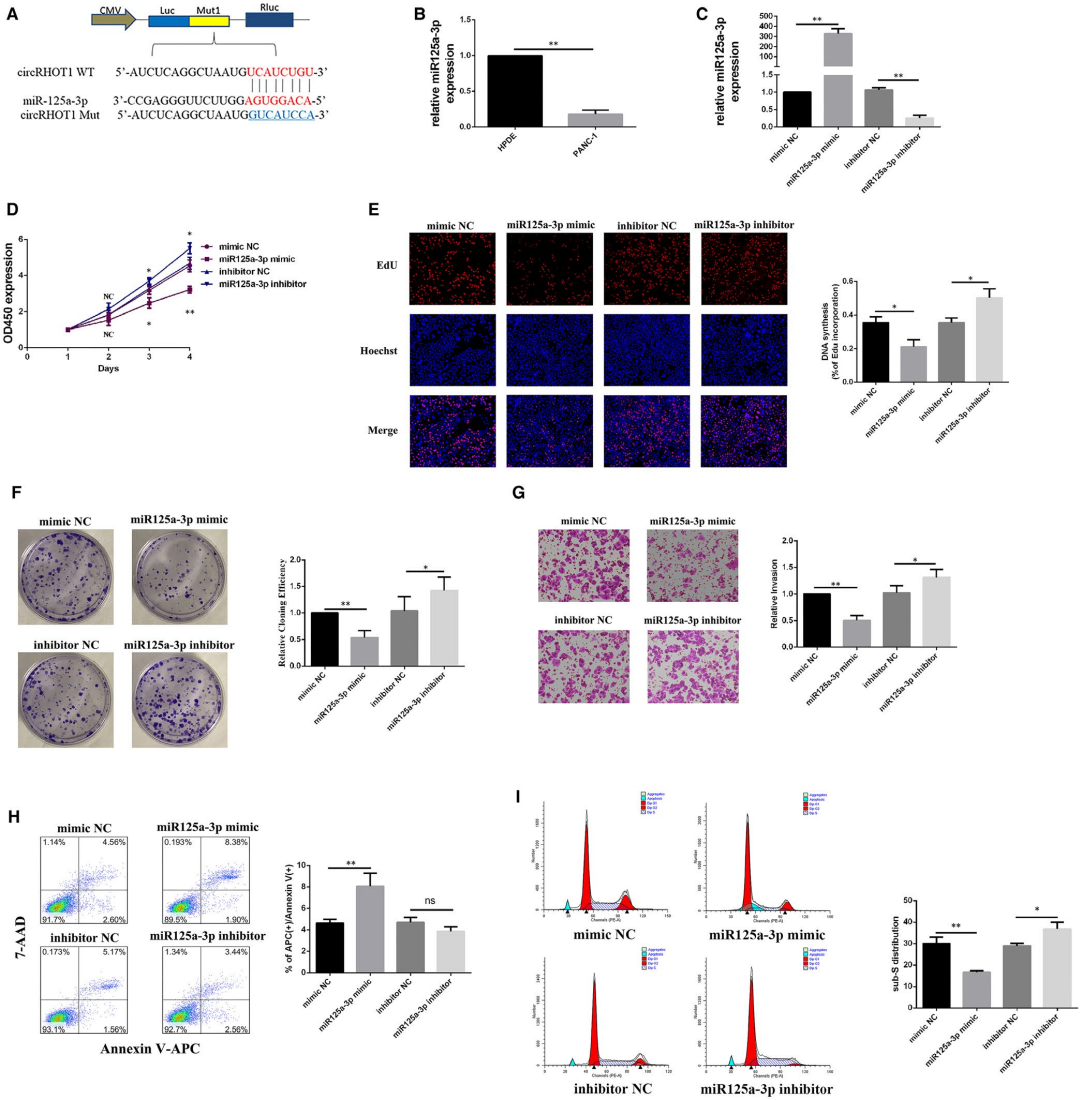
MiR‐125a‐3p is crucial for regulating the biological function of PANC‐1 cells. A, Putative complementary sites within circRHOT1 and miR‐125a‐3p were predicted by TargetScan. B, Relative expression of miR‐125a‐3pin human pancreatic ductal cell line (HPDE) and PANC‐1 cells was measured by RT‐qPCR. C, Relative expression levels of miR‐125a‐3p in PANC‐1 cells after transfection were measured by RT‐qPCR. D, Cell Counting Kit‐8 assays were used to detect the viability of PANC‐1 cells after transfection. E, 5‐Ethynyl‐20‐deoxyuridine assays were used to detect cell proliferation after transfection. F, Colony formation assays were used to detect clonogenic ability in PANC‐1 cells after transfection G, Transwell assays were used to detect cell invasion capacities in PANC‐1 cells after transfection. H and I, Flow cytometric assays were used to detect apoptosis and to assess the cell cycle after transfection. * P < .05, ** P < .01

By using TargetScan, miR‐125a‐3p was shown to have a binding site for circRHOT1 (Figure 3A). Then, the expression levels of miR‐125a‐3p in HPDE and PANC‐1 cells were examined by using RT‐qPCR. The results indicated that the expression level of miR‐125a‐3p in PANC‐1 cells was significantly decreased relative to that of HPDE cells (Figure 3B). To investigate the function of miR‐125a‐3p in PANC‐1 cells, a miR‐125a‐3p mimic and an inhibitor were used to regulate the expression of miR‐125a‐3p. The RT‐qPCR results showed the efficiency of the miR‐125a‐3p mimic and inhibitor (Figure 3C). Overexpression of miR‐125a‐3p reduced cell proliferation (Figure 3D,E), reduced the colony‐forming capacity (Figure 3F) and suppressed the invasive potential of PANC‐1 cells relative to the control cells (Figure 3G); however, the opposite was true for the miR‐125a‐3p inhibitor. In addition, flow cytometry demonstrated that up‐regulated miR‐125a‐3p promoted apoptosis (Figure 3H) and reduced the number of PANC‐1 cells arrested in S phase (Figure 3I). In contrast to the miR‐125a‐3p mimic group, decreased miR‐125a‐3p reduced the apoptosis rate (Figure 3H) and induced S phase arrest in PANC‐1 cells (Figure 3I).

### 3.4 CircRHOT1 regulates the expression of E2F3 by targeting miR‐125a‐3p in PANC‐1 cells

**FIGURE 4:**
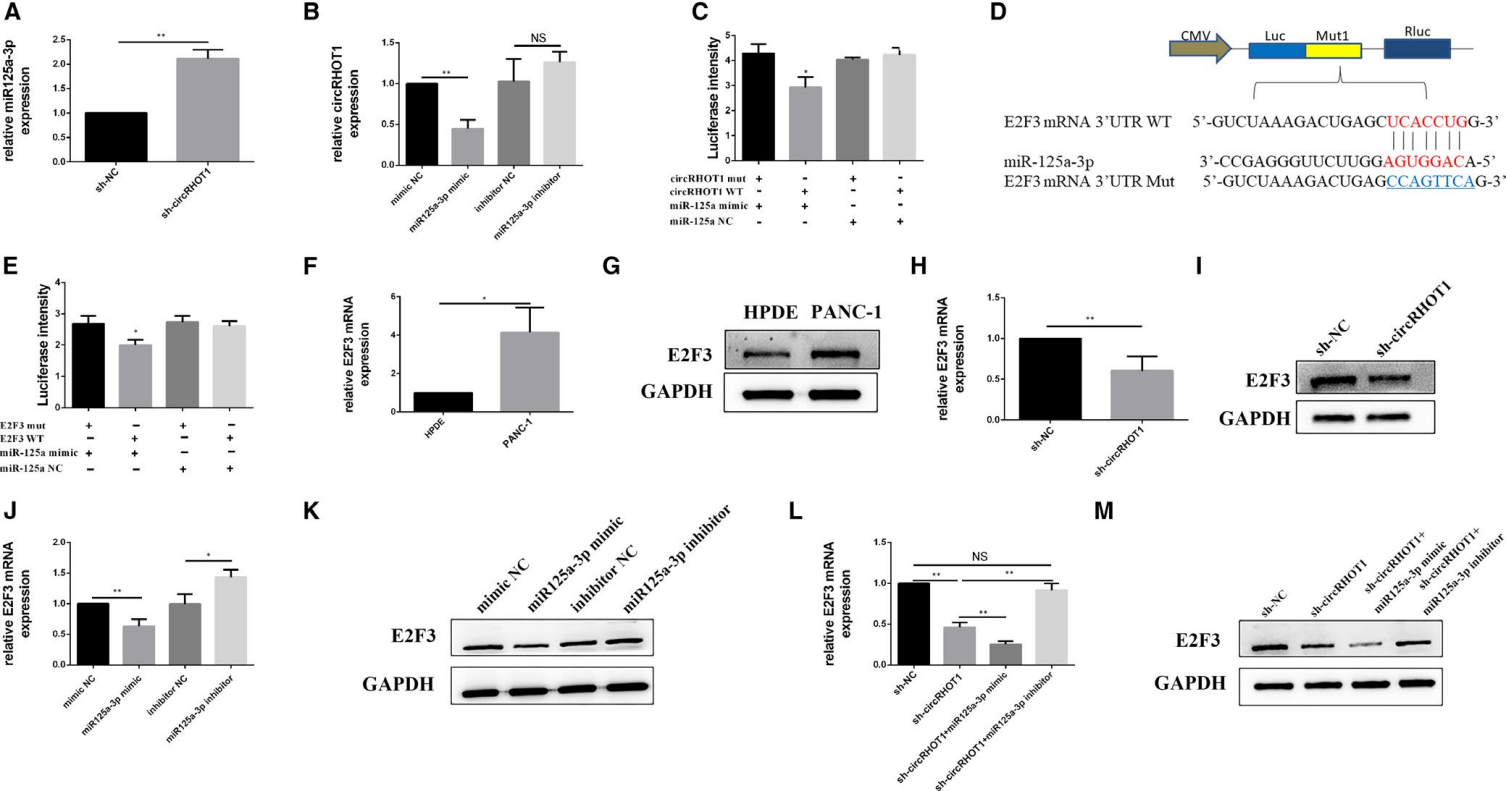
CircRHOT1 regulates the expression of E2F3 by targeting miR‐125a‐3p in PANC‐1 cells. A, RT‐qPCR was used to measure the relative expression of miR‐125a‐3pin PANC‐1 cells after transfection. B, RT‐qPCR was used to measure the expression level of circRHOT1 in PANC‐1 cells after transfection. C, Dual‐luciferase reporter assays were used to test the putative complementary sites within circRHOT1 with miR‐125a‐3p. D, Predicted binding sites of miR‐125a‐3p in the E2F3 3ʹUTRby TargetScan. E, Dual‐luciferase reporter assays were used to test the putative complementary sites within miR‐125a‐3p and the E2F3 3ʹUTR. F and G, Relative expression of E2F3mRNA and protein in human pancreatic ductal cell line (HPDE) and PANC‐1 cells was measured by RT‐qPCR. H and I, RT‐qPCR and Western blotting were used to measure the expression level of E2F3in PANC‐1 cells after circRHOT1 knockdown. J and K, Relative expression of E2F3mRNA and protein measured by RT‐qPCR and Western blot after transfection. L and M, Relative E2F3mRNA and protein expression was measured by RT‐qPCR and Western blot after transfection. * P < .05, ** P < .01

To confirm the relationship between circRHOT1 and miR‐125a‐3p, RT‐qPCR was used to detect the expression levels of miR‐125a‐3p and circRHOT1 after cells were treated with sh‐circRHOT1, a miR‐125a‐3p mimic or a miR‐125a‐3p inhibitor. The results indicated that circRHOT1 knockdown partly rescued the expression of miR‐125a‐3p (Figure 4A) and that circRHOT1 expression was negatively correlated with miR‐125a‐3p expression in PANC‐1 cells (Figure 4B). A luciferase reporter assay revealed that miR‐125a‐3phad binding sites for circRHOT1 (Figure 4C). The above results suggest that circRHOT1 directly targets miR‐125a‐3p and negatively regulates it.

The probable binding sites in the target genes of miR‐125a‐3p were also predicted using TargetScan. The results showed that the E2F3 3′‐UTR was strongly linked tomiR‐125a‐3p (Figure 4D), and the luciferase reporter assay confirmed this result (Figure 4E). Then, the expression of E2F3 in HPDE and PANC‐1 cells was examined by using RT‐qPCR and Western blot analysis. The results indicated that the expression of E2F3 in PANC‐1 cells was increased relative to that in HPDE cells (Figure 4F,G). The expression of E2F3 was decreased after PANC‐1 cells were treated with sh‐circRHOT1 (Figure 4H,I). Additionally, the expression of E2F3was negatively correlated with miR‐125a‐3p expression when cells were treated with a miR‐125a‐3p mimic and an inhibitor (Figure 4J,K). Moreover, RT‐qPCR data and Western blot analysis showed that the miR‐125a‐3p inhibitor could limit the decrease in E2F3 expression induced by sh‐circRHOT1 (Figure 4L,M).

### 3.5 E2F3 is involved in modulating the biological functions of PANC‐1 cells

**FIGURE 5:**
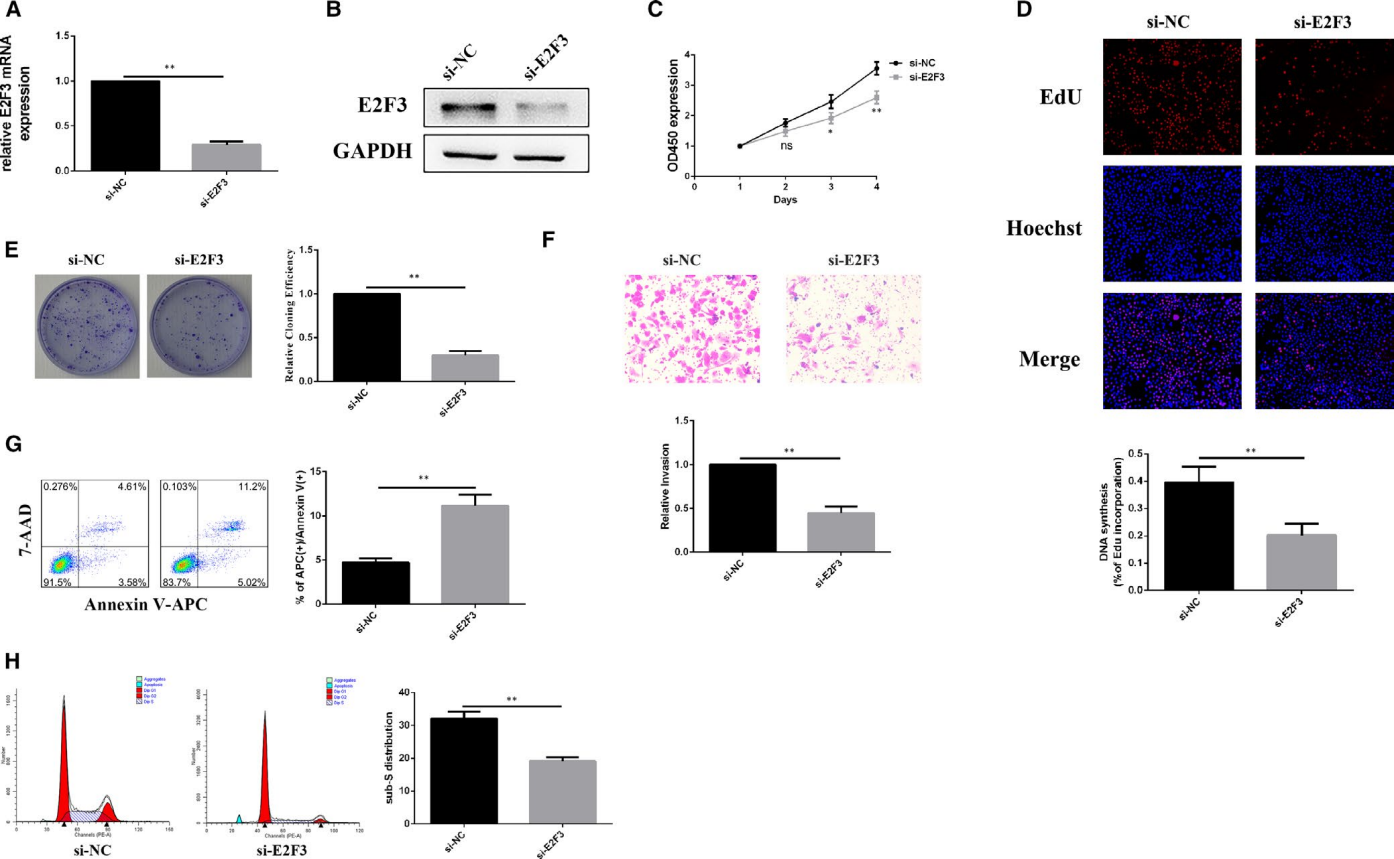
E2F3 is involved in modulating the biological function of PANC‐1 cells. A and B, Relative E2F3 expression was measured by RT‐qPCR and Western blot after E2F3 knockdown. C and D, Cell Counting Kit‐8 and 5‐ethynyl‐20‐deoxyuridine assays were used to detect the viability of PANC‐1 cells after transfection. E, Colony formation assays were used to detect colony‐forming capacity in PANC‐1 cells after transfection. F, Transwell assays were used to detect cell invasion capacities in PANC‐1 cells after E2F3 knockdown. G and H, Flow cytometric assays were used to detect apoptosis and to assess the cell cycle after E2F3 knockdown. * P < .05, ** P < .01

To confirm whether E2F3 could modulate the biological function of PANC‐1 cells, si‐E2F3 was used to knock down the expression of E2F3 in PANC‐1 cells. The RT‐qPCR and Western blot results showed that the expression of E2F3 was significantly decreased 48h after transfection (Figure 5A,B). The results from CCK‐8 and EdU assays showed that the viability of PANC‐1 cells was significantly decreased following treatment with si‐E2F3 (Figure 5C,D). E2F3 knockdown evidently suppressed the colony‐forming capacity of PANC‐1 cells (Figure 5E). Additionally, decreased E2F3 suppressed the invasive potential of PANC‐1 cells (Figure 5F). Finally, flow cytometry demonstrated that knockdown of E2F3 promoted apoptosis (Figure 5G) and reduced the number of PANC‐1 cells arrested in S phase (Figure 5H). The above results suggest that E2F3 is involved in modulating biological functions of PANC‐1 cells.

## 4 DISCUSSION

Our study demonstrates that circRHOT1 is overexpressed in PDAC tissues, while miR‐125a‐3p is down‐regulated. circRHOT1 is up‐regulated in different kinds of pancreatic cancer cell lines, especially PANC‐1 cells. Increased expression of circRHOT1 is correlated with lymphatic metastasis in PDAC and inhibition of miR‐125a‐3p with concomitant downstream up‐regulation of E2F3, leading to the proliferation and invasion of PANC‐1 cells. This study highlights the role of circRHOT1 in pancreatic cancer and highlights circRHOT1 as a potential diagnostic and therapeutic target.

Previous studies have shown that circRHOT1 is increased in pancreatic cancer tissues and cells.^14^ Further, circRHOT1 promotes proliferation and invasion of pancreatic cancer cells.^16^ In HCC, circRHOT1 is significantly up‐regulated and initiate NR2F6 expression to promote tumour progression, the expression of circRHOT1 was related to patient prognosis.^15^ CircRNAs have been regarded as regulators or biological markers in many types of diseases, including pancreatic cancer. The classic function of circRNAs is their acting as a miRNA sponge to regulate the expression of miRNA target genes. A previous study indicated that circ_0007534 regulates cell proliferation, apoptosis and invasion by sponging miR‐625 and miR‐892b in PDAC.^17^ Another study suggested that circ_0006215 could regulate the expression of SERPINA4 by targeting miR‐278a‐3p in pancreatic cancer cells.^18^ Our study shows a novel regulatory relationship between circRHOT1 and miR‐125a‐3p in pancreatic cancer. It has been reported that the expression of miR‐125 is decreased in numerous kinds of cancer.^19^ A recent study showed that miR‐125asuppresses colorectal cancer progression by targeting VEGFA.^20^ miR‐125a suppresses the progression of bladder cancer by targeting FUT4.^21^ These results suggest that miR‐125a might serve as a tumour suppressor in cancers. In our study, miR‐125a‐3p was decreased in PANC‐1 cells, and overexpression of miR‐125a‐3p reduced cellular proliferation and invasion and promoted apoptosis in PANC‐1 cells. The qPCR and luciferase reporter assay results indicated that miR‐125a‐3p is a downstream target of circRHOT1. In addition, knockdown of miR‐125a‐3p could stifle the biological functions induced by inhibiting circRHOT1. These results suggest that circRHOT1 modulates biological functions of PANC‐1 cells via regulation of miR‐125a‐3p.

TargetScan was employed to identify the downstream targets of miR‐125a‐3p, and E2F3 was selected for further analysis. Our current study indicates that the miR‐125a‐3p/E2F3 axis plays a critical role in the proliferation, apoptosis and invasion of PANC‐1 cells. E2F3 is a member of the E2F family and serves as a transcription factor that could play an important role in cell proliferation, apoptosis and so on.^22^ Numerous studies have shown that E2F3 functions in the control of tumour progression and is increased in different kinds of cancers.^23^, ^24^, ^25^, ^26^Consistent with previous studies, the present study found that E2F3 was increased in PANC‐1 cells. Additionally, knockdown of E2F3 could reduce proliferation and invasion and promote apoptosis of PANC‐1 cells. The qPCR and Western blot results indicated that miR‐125a‐3p could regulate the expression of E2F3, and the luciferase reporter assay results showed that miR‐125a‐3p mimics obviously decreased the luciferase activity of E2F3‐WT. Taken together, these data suggest that E2F3 acts as a direct target of miR‐125a‐3p. Our data showed that circRHOT1 inhibition decreased E2F3 expression, which could be reversed by treatment with miR‐125a‐3p inhibitors. These results suggest that circRHOT1 performs biological functions in PANC‐1 cells via regulation of the miR‐125a‐3p/E2F3 axis. However, our study has several limitations. Owing to the difficulty in collecting pancreatic cancer samples, the number of samples is low. Further study with a large sample size is needed to confirm these findings.

## 5 CONCLUSIONS

Our data reveal that circRHOT1 is increased in PDAC tissues and cell lines. Additionally, circRHOT1 was correlated with lymphatic metastasis in PDAC, and it was found to regulate the proliferation, invasion and apoptosis of pancreatic cells by down‐regulating miR‐125a‐3p to increase the expression of E2F3. Therefore, circRHOT1 might be a reasonable diagnostic and therapeutic target for pancreatic cancer.

## CONFLICT OF INTEREST

The authors declare that there are no potential conflicts of interest.

## AUTHOR CONTRIBUTION

Sunkai Ling: Methodology (equal); Writing‐original draft (equal). Yanru He: Formal analysis (equal); Resources (equal); Validation (equal). Xiaoxue Li: Formal analysis (equal); Writing‐review & editing (equal). Mingyue Hu: Formal analysis (equal); Resources (equal). Yu Ma: Formal analysis (equal); Writing‐review & editing (equal). Yuan Li: Formal analysis (equal). Zipeng Lu: Resources (equal). Shanshan Shen: Writing‐review & editing (equal). Bo Kong: Writing‐review & editing (equal). Xiaoping Zou: Writing‐review & editing (equal). Kuirong Jiang: Resources (equal). Peilin Huang: Conceptualization (equal); Data curation (equal); Funding acquisition (equal).

## CONSENT FOR PUBLICATION

All authors gave final approval of the version to be published and agree to be accountable for all aspects of the work.

## ETHICS APPROVAL AND CONSENT TO PARTICIPATE

The experiments utilizing human samples were approved by the Ethical Committee of Medical Research, the Affiliated Drum Tower Hospital of Nanjing University Medical School (2016‐191‐01).

## Data Availability

All data, models or code generated or used during the study are available from the corresponding author by request.
